# Combined lentiviral- and RNA-mediated CRISPR/Cas9 delivery for efficient and traceable gene editing in human hematopoietic stem and progenitor cells

**DOI:** 10.1038/s41598-020-79724-x

**Published:** 2020-12-28

**Authors:** David Yudovich, Alexandra Bäckström, Ludwig Schmiderer, Kristijonas Žemaitis, Agatheeswaran Subramaniam, Jonas Larsson

**Affiliations:** Division of Molecular Medicine and Gene Therapy, Lund Stem Cell Center, Lund University, BMC A12, 221 84 Lund, Sweden

**Keywords:** Genetic engineering, Haematopoietic stem cells

## Abstract

The CRISPR/Cas9 system is a versatile tool for functional genomics and forward genetic screens in mammalian cells. However, it has been challenging to deliver the CRISPR components to sensitive cell types, such as primary human hematopoietic stem and progenitor cells (HSPCs), partly due to lentiviral transduction of Cas9 being extremely inefficient in these cells. Here, to overcome these hurdles, we developed a combinatorial system using stable lentiviral delivery of single guide RNA (sgRNA) followed by transient transfection of Cas9 mRNA by electroporation in human cord blood-derived CD34^+^ HSPCs. We further applied an optimized sgRNA structure, that significantly improved editing efficiency in this context, and we obtained knockout levels reaching 90% for the cell surface proteins CD45 and CD44 in sgRNA transduced HSPCs. Our combinatorial CRISPR/Cas9 delivery approach had no negative influence on CD34 expression or colony forming capacity in vitro compared to non-treated HSPCs. Furthermore, gene edited HSPCs showed intact in vivo reconstitution capacity following transplantation to immunodeficient mice. Taken together, we developed a paradigm for combinatorial CRISPR/Cas9 delivery that enables efficient and traceable gene editing in primary human HSPCs, and is compatible with high functionality both in vitro and in vivo.

## Introduction

Due to its simplicity and adaptability, the CRISPR/Cas9 system has rapidly become one of the most popular approaches for genome engineering and is accelerating the study of many biological systems. Genetic perturbations by the CRISPR/Cas9 system can be applied either as targeted perturbations looking to identify the function of a single gene^1,2^ or in a wider context with the application of genetic screens^3,4^. Lentiviral marking of cells can extend the application of the CRISPR system towards multiplexed perturbations^5–7^ and cell tracking^8,9^. In a wide variety of mammalian cell types, the CRISPR system has proven straightforward and robust when delivered through a single lentiviral vector^6,10,11^. However, lentiviral delivery of the CRISPR components to primary human hematopoietic stem and progenitor cells (HSPCs) has proven challenging and limited the applications of CRISPR/Cas9 in these cells^12,13^. Recently, several groups have shown that ribonucleoproteins (RNPs), *Streptococcus pyogenes* Cas9 (Cas9) protein in complex with single guide RNA (sgRNA), can be used to obtain efficient genome editing in human HSPCs when delivered by electroporation^14–16^. In addition, it has been reported that modified sgRNAs enhance genome editing in CD34^+^ HSPCs, demonstrating an advantage over unmodified sgRNAs in human cells when co-delivered with Cas9 mRNA or delivered as RNP complex due to their increased stability^17^. Yet, these approaches do not allow tracing of the edited cells. The delivery of sgRNAs by stable lentiviral transduction would be advantageous as it can be coupled with expression of a marker gene for cell tracking and cell sorting. Moreover, in the context of multiplexed editing and pooled screens, it is possible to monitor the distribution of integrated proviral sgRNAs by next generation sequencing (NGS).

Previously, we have reported on the usefulness and relevance of shRNA lentiviral screens in the human hematopoietic system^18–21^ and considered the applicability of CRISPR screens to HSPCs. In this study, we sought to solve the lentiviral delivery challenge of the CRISPR/Cas9 system in human HSPCs by combining lentiviral sgRNA transduction with transient delivery of Cas9 mRNA by electroporation. We successfully introduced this combined strategy and, further, implemented a second-generation chimeric guide RNA backbone which improved the editing efficiency in HSPCs. This method is efficient, modular, cost-effective and importantly, compatible with high HSPC functionality in vitro and in vivo. As such, we propose a new optimal way for integrated gene editing work in HSPCs suitable for lentiviral combinatorial perturbations, cell tracking, and genome-wide screens.

## Results

### Challenge of lentiviral Cas9 delivery in primary human HSPCs

**Figure 1:**
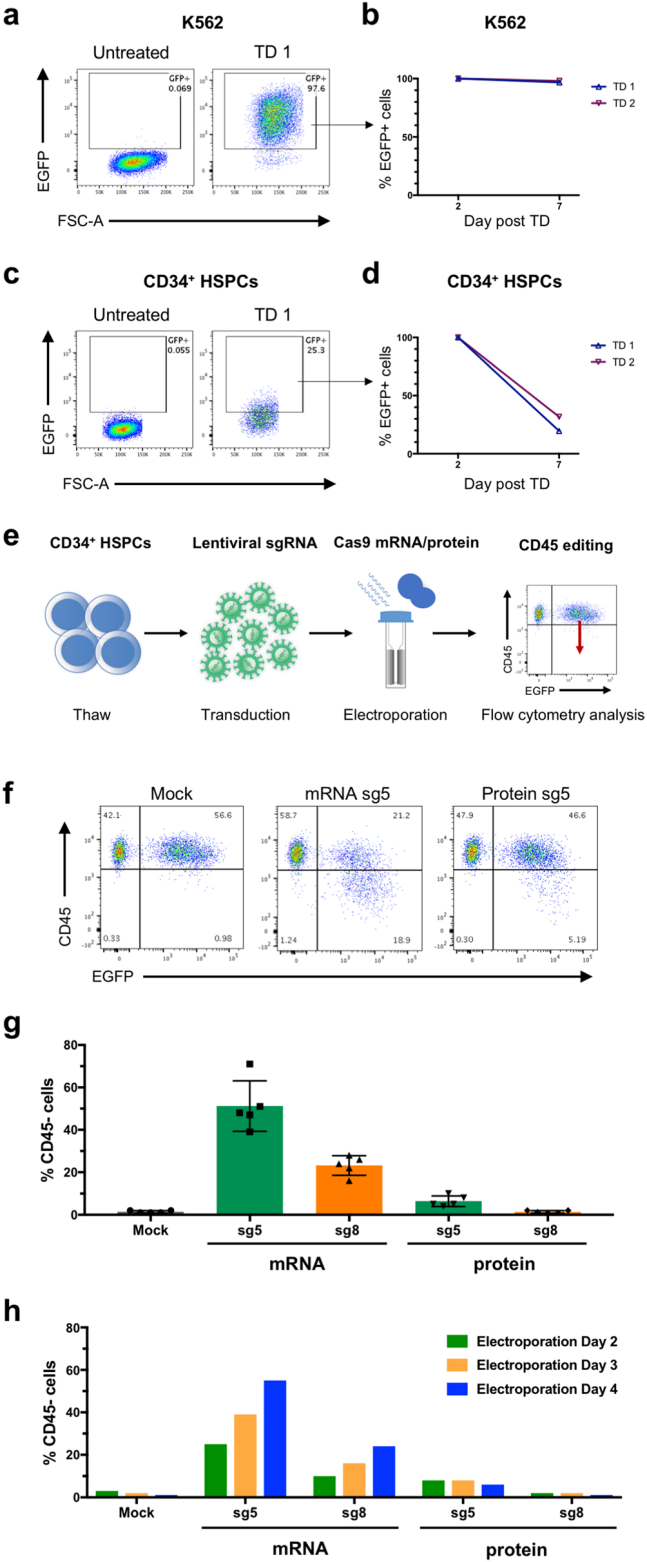
CRISPR editing in human HSPCs using lentiviral sgRNA and Cas9 mRNA or protein. (**a**) Transduction efficiency of K562 cells treated with lentivirus carrying full-length Cas9.P2A.EGFP, as determined by flow cytometry on day 7. (**b**) Maintenance of EGFP expression following transduction and EGFP sorting of K562 cells. Data from two independent transductions (TD 1 and TD 2) (**c**) Transduction efficiency of CD34^+^ HSPCs, treated with lentivirus carrying full-length Cas9.P2A.EGFP, as determined by flow cytometry on day 7. (**d**) Maintenance of EGFP expression following transduction and EGFP sorting of CD34^+^ HSPCs. Data from two independent transductions (TD 1 and TD 2) (**e**) Overview of experimental outline for gene editing in CD34^+^ HSPCs using lentiviral delivery of sgRNA targeting CD45 and electroporation of Cas9 mRNA or protein. (**f**) Representative FACS plots showing CD45 editing in primary CD34^+^ HSPCs following lentiviral delivery of sgRNA (sg5) and electroporation of Cas9 mRNA or protein. (**g**) Efficiency of CD45 editing in CD34^+^ HSPCs following lentiviral delivery of sgRNA (sg5 or sg8) and electroporation of Cas9 mRNA or protein (*n* = *5*). (**h**) The effect of time in culture after transduction prior to Cas9 mRNA or protein electroporation on CD45 editing efficiency. All samples were transduced on day 1 with sg5 or sg8, electroporated on either day 2, 3 or 4, and subsequently analysed by flow cytometry 4 days following the electroporation.

Given the opportunities of CRISPR/Cas9 gene editing for functional modelling and genetic screens, we assessed lentiviral Cas9 delivery to primary human cord blood (CB)-derived HSPCs. We adapted the all-in-one Cas9 pLentiCRISPRv2 (pLCv2) vector with a P2A.EGFP cassette for work in cell lines and HSPCs. Vector transduction in the human leukaemia cell line K562 was successful with stable, long term EGFP expression (Fig. 1a,b). However, the transduction efficiency in CD34^+^ HSPCs was very low and expression of the vector EGFP was lost over time (Fig. 1c,d). Upon testing of several backbones, promoters, and multiplicities of infection (MOI), the Cas9 containing backbones consistently failed to robustly express EGFP (Supplementary Fig. S1). Interestingly, this effect was seen even upon the expression of a truncated form of Cas9 (half) or upon the expression of the smaller Cas9 variant from *Staphylococcus aureus* (SaCas9), while other similarly sized vectors without Cas9 (insert approx. 4 kb) demonstrated robust and stable EGFP expression (Supplementary Fig. S1).

Taken together, we demonstrate that lentiviral Cas9 delivery in human HSPCs is challenging both in terms transduction efficiency and stable expression, and that this a function of the Cas9 protein itself, rather than vector parameters or transduction conditions.

### Lentiviral sgRNA combined with Cas9 protein or mRNA promote HSPC editing

To address the lentiviral expression problem of Cas9, we turned towards other delivery methods to express Cas9 in HSPCs. Recently, both protein and mRNA electroporation of Cas9 together with synthetic sgRNAs has been successfully demonstrated in HSPCs^15,16^. Following comparison of different electroporation methods, we chose to use the BTX ECM 830 electroporation system, which in our hands demonstrated good transfection efficiency and reasonable toxicity in primary HSPCs. Indeed, similar to previous reports, we found that electroporation of both Cas9 protein and mRNA together with synthetic sgRNAs were highly functional methods for editing of the pan hematopoietic marker CD45 (*PTPRC* gene) in CD34^+^ HSPCs as illustrated by flow cytometry analysis (Supplementary Fig. S2). However, this approach does not allow for tracking of specific perturbations or multiplexed gene editing in a pooled fashion, since the manipulation is transient without any permanent marking of the cells. Therefore, we reasoned that combining transient expression of Cas9 with lentiviral delivery of sgRNA could be a possible alternative for retaining guide-based cell marking by vector integration and for tracking functionality.

Next, we tested such split delivery of electroporated Cas9 and lentiviral sgRNA (Fig. 1e). Specifically, we transduced cells with sgRNAs targeting CD45 using a lentiviral vector co-expressing EGFP, and introduced Cas9 either as protein or mRNA by electroporation at concentrations we had previously determined to be optimal for editing efficiency and viability (Supplementary Fig. S2). We found that the editing, although less efficient than with synthetic sgRNAs, was successful in both the context of Cas9 mRNA and protein (Fig. 1f,g, Supplementary Fig. S3). However, unlike the situation with transiently delivered synthetic sgRNAs, we observed a markedly higher editing efficiency in Cas9 mRNA edited samples compared to those treated with Cas9 protein at the given concentrations. This could relate to the available intracellular Cas9 concentration being sustained for a longer time with mRNA delivery and thus creating a broader editing window to functionally interact with the stably expressed sgRNA, or that higher protein levels are required in this context.

Next, we assessed whether our combined delivery method showed different off-target activity compared to the established Cas9 RNP or Cas9 mRNA with synthetic sgRNA. We analyzed four predicted off-target sites for sg5 targeting CD45 using NGS. We found an overall low off-target activity both for synthetic and lentiviral sgRNA delivery except for OT site #2 where the RNP method showed a relatively high frequency of off-target modification (10%) (Supplementary Table 1 and 2). Importantly, these findings indicate that our combined delivery method does not have a higher propensity for off-target events compared to other established methods for gene editing in CD34^+^ HSPCs.

Previously, it has been reported that culture time can affect the efficiency of gene editing^15^. To test for the effect of the time in culture prior to electroporation on the editing efficiency, cells were transduced 24 h after thawing and thereafter electroporated at 24-h intervals to evaluate the optimal editing time. Interestingly, we saw that the editing efficiency improved with increased time between transduction and Cas9 delivery (Fig. 1h, Supplementary Fig. S3). One explanation for this is that there may be a delay until maximal amounts of sgRNA are expressed from the lentiviral vector. Stem cell potential is known to be gradually lost in culture and we consequently observed lower CD34^+^ cell frequency with later Cas9 delivery (Supplementary Fig. S3). It remains to be determined under which condition functional stem cells are most efficiently edited. For the subsequent experiments, we chose to deliver Cas9 72 h after sgRNA transduction.

In summary, we have developed a paradigm for successful editing of primary human HSPCs with integrated, trackable lentiviral sgRNA vectors in combination with Cas9 mRNA or protein.

### An optimized sgRNA structure enhances gene editing efficiency in primary HSPCs

**Figure 2:**
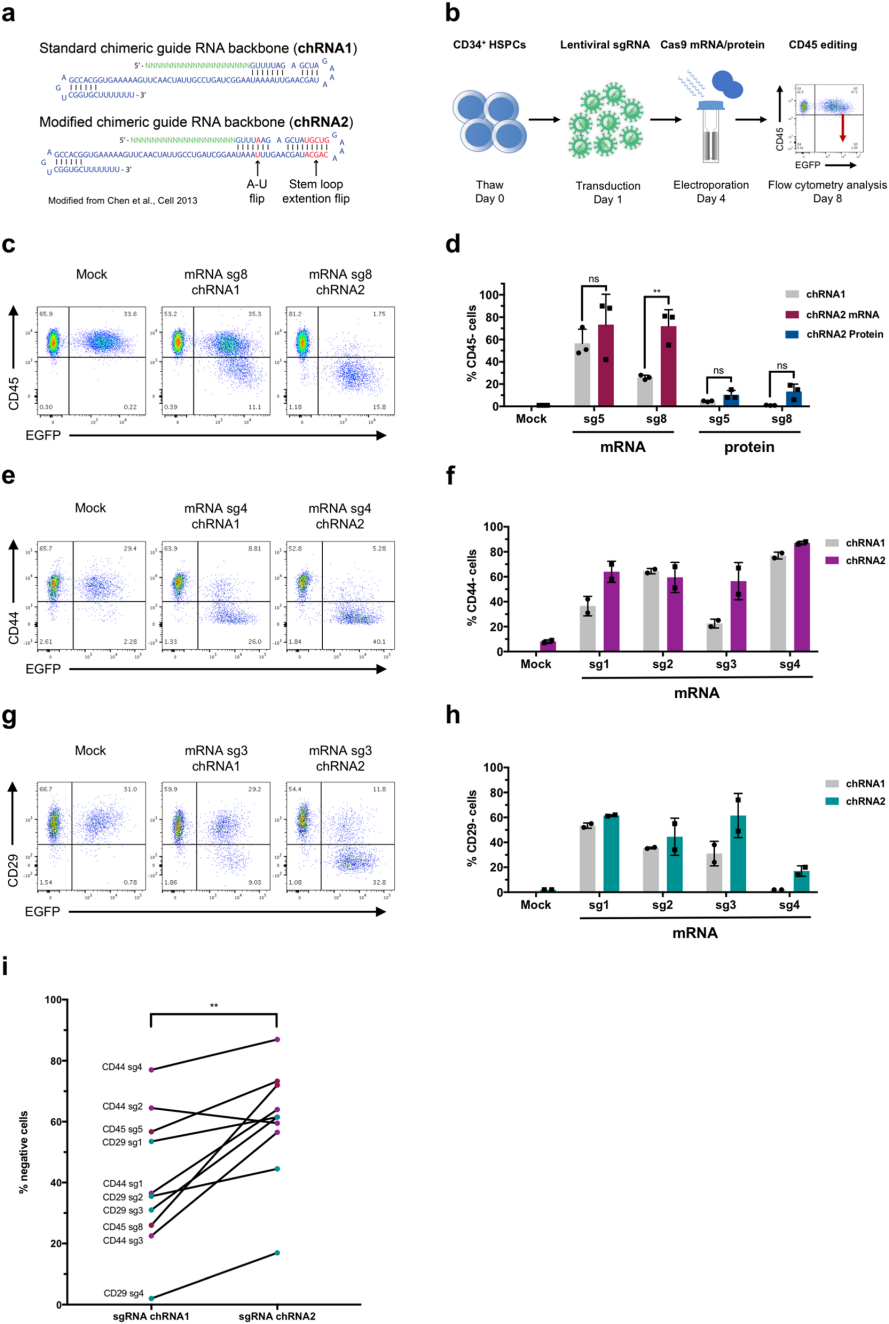
Modified chimeric guide RNA backbone enhances editing efficiency in HSPCs. CD34^+^ HSPCs were transduced with lentivirus expressing sgRNAs encoding chimeric guide RNA backbone 1 (chRNA1) or 2 (chRNA2) on day 1, followed by electroporation of Cas9 mRNA or protein on day 4. Editing was then assessed by flow cytometry 4 days post-electroporation on day 8. (**a**) Overview of structural changes between the standard chimeric guide RNA backbone 1 (chRNA1) versus the modified chimeric guide RNA backbone 2 (chRNA2). This figure is modified from Chen et al.^8^ (**b**) Overview of optimized experimental outline for comparing chimeric guide RNA backbone configurations. (**c**) Representative FACS plots of CD45 editing. (**d**) CD45 editing efficiency assessed by flow cytometry. Statistical significance was calculated using unpaired student’s t-test. **p = 0.0058. (**e**) Representative FACS plots of CD44 editing. (**f**) CD44 editing efficiency. (**g**) Representative FACS plots of CD29 editing. (**h**) CD29 editing efficiency. (**i**) Comparison of editing efficiency between chRNA1 and chRNA2 in terms of % negative cells, for different sgRNAs with different gene targets (CD45, CD44, CD29), measured by flow cytometry 4 days post-electroporation. Statistical significance was calculated using paired student’s t-test. **p = 0.0031. Error bars represent standard deviation (s.d.).

Given the relatively lower efficiency of our combined transduction/electroporation treatment, compared to our results with synthetic, modified sgRNAs, we sought to improve our method in terms of editing efficiency. Specifically, we hypothesized that the difference in efficiency could be due to a lower abundance of sgRNA in the lentiviral context compared to electroporated amounts. Therefore, we explored strategies for enhancing intracellular sgRNA function and expression. Previously, Chen et al.^8^ and Dang et al.^22^ reported on optimizations to the sgRNA structure that improved CRISPR function and editing. However, as these optimizations have not been broadly implemented, it was not clear if they would be beneficial for editing in primary HSPCs and in the context of electroporated Cas9 mRNA or protein. We adopted these improvements into a new sgRNA structure, which we denote as the second-generation chimeric guide RNA backbone or in short chRNA2 (Fig. 2a).

To assess the utility of the chRNA2 in CB-derived CD34^+^ HSPCs, we transduced cells with lentiviral sgRNA targeting CD45 encoding the original (chRNA1) or modified (chRNA2) backbone structure, followed by our optimized electroporation with Cas9 mRNA or protein to explore if the editing efficiency could be enhanced (Fig. 2b). We found that the chRNA2 improved the editing efficiency for the sgRNAs targeting CD45 compared to the original chRNA1 structure, in conditions treated with both Cas9 mRNA and protein (Fig. 2c,d). Interestingly, when using the chRNA2 structure, we saw a significantly increased editing efficiency by the less effective sgRNA8 (sg8), which reached a comparable editing level to that of the highly efficient sgRNA5 (sg5). In addition, a trend of increased editing using the chRNA2 compared to the original chRNA1 was seen for sg5 using both Cas9 mRNA and protein, although the difference was not significant. Overall viability and frequency of CD34^+^ cells were similar between the different conditions (Supplementary Fig. S4). This indicates that sgRNA performance can be boosted by implementing the chRNA2 structure, particularly with underperforming sgRNAs.

To further validate the performance of chRNA2, we repeated the comparison with additional lentiviral sgRNAs targeting the highly expressed cell surface proteins CD44 and CD29 using Cas9 mRNA. We observed a similar trend of the second-generation guide RNA backbone markedly increasing the editing efficiency for several individual sgRNAs tested (Fig. 2e–h, Supplementary Fig. S4). When comparing editing efficiency results between flow cytometry and NGS analyses, we observed a higher editing efficiency by NGS, in particular for CD29. This could partially be due to monoallelic editing which will not be detected by flow cytometry or high protein stability such that the CD29 protein expression is retained and detected by flow cytometry for a period of time despite successful editing.

Finally, we compared the use of the original chRNA1 to the modified chRNA2 structure for all the tested gene targets (CD45, CD44 and CD29) and sgRNAs (Fig. 2i). This comparison shows that the chRNA2 structure significantly increases editing efficiency across several genetic loci and multiple guides.

In conclusion, the chRNA2 sgRNA structure is an important improvement to our combined lentiviral- and RNA-mediated CRISPR/Cas9 approach that enhances the editing efficiency.

### Human HSPCs show preserved stem and progenitor cell function following combined lentiviral- and RNA-mediated CRISPR/Cas9 gene editing

**Figure 3:**
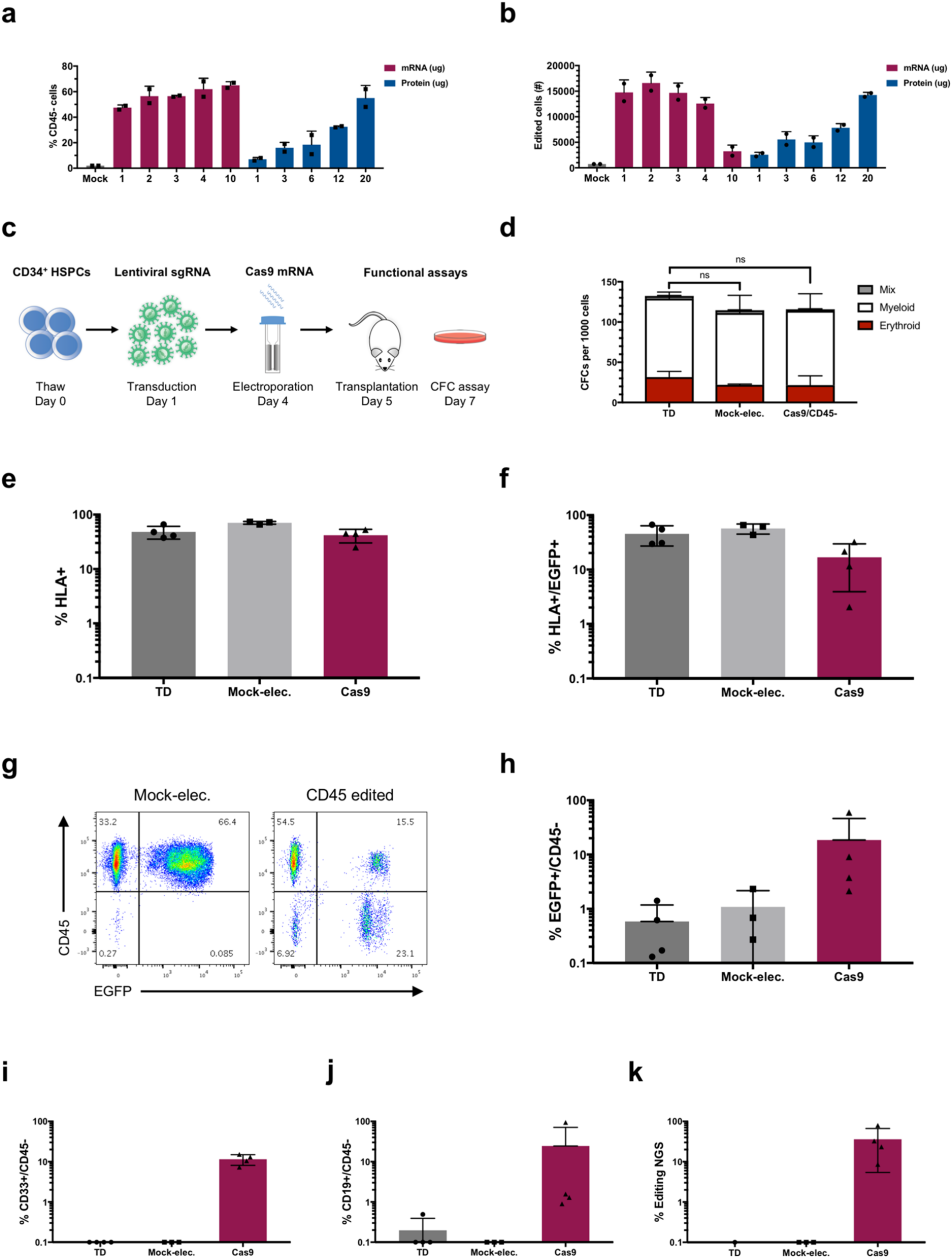
Edited CD34^+^ HSPCs retain colony forming capacity in vitro and reconstitution capacity in vivo. (**a,b**) Primary CD34^+^ cells were transduced with sgRNA for CD45 (sg8, chRNA2) on day 1, electroporated with increasing amounts of Cas9 mRNA or protein on day 4, and subsequently analysed by flow cytometry on day 8 for CD45 expression. Data presented as (**a**) frequency of CD45 edited cells in the EGFP^+^ population, or (**b**) total number of CD45 edited cells retrieved from each condition. (**c**) Overview of experimental outline for evaluating CD34^+^ HSPC functionality. CD34^+^ HSPCs were transduced with sgRNA for CD45 (sg8, chRNA2) and electroporated with 2.5 μg Cas9 mRNA per 100 k cells (Cas9), mock-electroporated (Mock-elec.) or kept not electroporated (TD), and subsequently transplanted into NSG mice one day later (day 5) or sorted for colony forming cell (CFC) assays on day 7. (**d**) CFC analysis per 1000 cells plated after sorting. Statistical significance for the total number of colonies for each group compared to TD cells was calculated using two-way ANOVA test. (**e-k**) NSG mice were transplanted with the equivalent of 100 k thawed CD34^+^ cells at the start of the experiment for the electroporated groups and half that dose (50 k cells) for the transduced, non-electroporated group. Bone marrow (BM) was harvested 9–12 weeks post-transplantation and cells were analysed using flow cytometry and NGS (n = 3–4 mice per group). **(e)** Frequency of human (HLA^+^) cells in BM. (**f**) Frequency of EGFP^+^ cells within the human HLA^+^ population. (**g**) Representative FACS plots showing EGFP and CD45 expression in the HLA^+^, CD19^+^ and CD33^+^ populations respectively of mice transplanted with mock-electroporated or Cas9-electroporated cells. (**h**) Frequency of edited CD45^-^ cells within the HLA^+^EGFP^+^ population. (**i**) Frequency of edited CD45^-^ cells within the myeloid HLA^+^EGFP^+^CD33^+^ population. (**j**) Frequency of edited CD45^-^ cells within the lymphoid HLA^+^EGFP^+^CD19^+^ population. (**k**) CD45 editing by NGS in sorted EGFP^+^ cells.

So far, we had observed successful editing with electroporation of both Cas9 mRNA and Cas9 protein and during the optimizations we considered both protein and mRNA reagents for future steps. Next, with a defined and optimized chRNA2 context, we aimed to compare the efficiency of Cas9 protein to mRNA. We tested a broad titration curve of Cas9 concentrations and analysed both the frequency of CD45- cells (Fig. 3a), as well as absolute numbers of edited cells (Fig. 3b), taking into account a dose dependent toxicity observed with high amounts of Cas9 mRNA. An optimal absolute editing efficiency was seen using 2 µg of Cas9 mRNA. Although 20 µg of Cas9 protein could reach similar efficiencies, this amount of protein is not practical to use in larger scale experiments due to high costs. Cas9 mRNA, on the other hand, can be easily synthesized in-house in large amounts and at a low cost. We therefore reasoned that Cas9 mRNA is better suited for gene editing work in the context of lentiviral sgRNA considering cost, scalability, and modularity in future HSPC applications.

To further investigate the functional utility of our method in primary human HSPCs, we used established assays to assess both progenitor cell function and stem cell potential (Fig. 3c). We first assayed CD45 edited cells for their colony forming ability in methylcellulose medium. Cas9 edited CD45^-^ cells were sorted and compared to transduced, non-electroporated cells and transduced, mock-electroporated cells. We saw no significant difference between the colony forming capacity of mock-electroporated and Cas9 edited CD45^-^ cells compared to the transduced, non-electroporated cells (Fig. 3d), indicating that the manipulation of the cells and the editing of CD45 is compatible with an intact ability to form colonies of the different lineages.

Finally, to assess the functionality of gene edited cells in terms of their stem cell potential and regenerative capacity in vivo, we performed a transplantation experiment using immunodeficient NOD Cg-Prkdc^scid^ Il2rg^tm1Wjl^/SzJ (NSG) mice as recipients. Equivalent cell doses of Cas9-electroporated or mock-electroporated sgCD45 transduced CB-derived CD34^+^ cells were transplanted into sublethally irradiated NSG mice. Half the equivalent dose of transduced non-electroporated cells was used as reference to compensate for previously demonstrated generic electroporation toxicity^15^. A portion of Cas9 treated cells maintained in culture showed a mean frequency of EGFP expression of 45%, and a mean CD45 editing efficiency of 67% within the EGFP positive population, as measured by flow cytometry analysis (Supplementary Fig. S5).

Bone marrow (BM) was analysed 9–12 weeks post-transplantation for reconstitution. Human engraftment levels as measured by the frequency of HLA positive cells were similar between the groups (Fig. 3e, Supplementary Fig. S5). We further detected robust EGFP expression for all the groups albeit at a lower frequency for the Cas9-electroporated group (Fig. 3g). Successful editing of CD45 was clearly detected within the EGFP positive population in both myeloid and lymphoid cells (Fig. 3f,h–k; Supplementary Fig. S5). However, the detected editing efficiency was lower than that observed in the non-transplanted cells. Together with the overall lower EGFP expression, this may be associated with the editing event inducing a DNA-damage response that negatively impacts HSCs. Alternatively, it cannot be excluded that the actual loss of CD45 would have an impact on the engraftment efficiency.

Nevertheless, our results clearly demonstrate that our editing strategy is compatible with robust and traceable editing of primary human HSCs with repopulation potential.

## Discussion

Here, we report on the development of a hybrid system, combining traceable lentiviral sgRNA delivery with transient delivery of Cas9 for CRISPR/Cas9 genome editing in primary human HSPCs. By delivering Cas9 as mRNA and using a modified sgRNA backbone, chRNA2^8,22^, we show that this approach is highly flexible and scalable, allowing for efficient editing with low toxicity and preserved stem and progenitor cell potential.

While the CRISPR/Cas9 system offers great opportunities for functional genetic studies in mammalian cells^7,23^, it has been challenging to exploit the full potential in human HSPCs due to the problems in delivering Cas9 by lentiviral transduction^12,13^. This could potentially be associated with the large size of the SpCas9 gene^24^ or a DNA damage response induced by the editing events^25^. Yet, we observed similar transduction problems using a smaller, truncated version of SpCas9 or the smaller SaCas9 variant, indicating that elements of the Cas9 protein itself are poorly tolerated by primary human HSPCs.

Our strategy to separately deliver the sgRNA component by lentiviral transduction is advantageous in a pooled screen context where an integrated and trackable component is required. However, the approach is also very useful for studying individual genes as the sgRNA transduced EGFP^+^ cells will be highly enriched for edited cells and can be directly compared to completely non-edited EGFP^-^ counterparts in functional assays of primary HSPCs, allowing high sensitivity with internally controlled experiments. Moreover, in experiments where targeting of multiple genes is desired, separate sgRNA vectors with distinct fluorescent markers can be co-transduced and tracked by Fluorescence Activated Cell Sorting (FACS).

Recently, two similar strategies using split delivery of Cas9 and sgRNA were reported. These studies used lentiviral sgRNA in combination with electroporation of Cas9 protein, either alone^26^, or in complex with non-targeting sgRNA forming RNPs^27^. Both these methods were applied to CRISPR screens in primary hematopoietic cells, including T cells and CD34^+^ cells, demonstrating the feasibility of such hybrid system in that context. While conceptually quite similar, our approach differs from these studies in several important aspects.

Firstly, we use Cas9 mRNA rather than protein, and we see several advantages, one being that mRNA is easy to produce at high quantities and is therefore very scalable. Moreover, the use of mRNA is extremely flexible as alternative Cas9 variants or other modifications, such as dCas9 variants or drug-inducible Cas9, can be swiftly implemented. There is further a significant cost–benefit as protein is currently expensive to obtain from commercial vendors and less trivial to produce in house. Only at very high amounts of Cas9 protein (20 μg per reaction) could we achieve similar editing as with mRNA. Such amounts of protein for screens or large-scale experiments would be challenging to produce and very expensive to purchase. The relatively higher efficiency of Cas9 mRNA compared to protein, observed here, could possibly be due to more durable availability in the cell by the continuous translation into Cas9 protein, thereby creating a larger time-window for interaction with the stably expressed sgRNA.

Secondly, we found that guide performance could be improved using a second-generation chimeric backbone structure for the sgRNA (chRNA2). The exact mechanism is unclear, but it has been suggested that chRNA2 affords higher levels of sgRNA expression compared to the original structure^22^. Yet, further studies are needed to confirm how this structure improves guide performance, especially of sub-optimal guides. Our findings argue for the use of chRNA2 for optimal guide performance, which is particularly important in screening contexts where efficient sgRNAs cannot be pre-selected.

Finally, our study is the first to evaluate aspects of functionality and stem cell potential in primary HSPCs subjected to CRISPR/Cas9 gene editing using a combined lentiviral and mRNA/protein delivery approach. When performing gene editing in primary cells, aspects of toxicity need to be carefully considered. The most pronounced toxicity in our hands was due to the electroporation. This transfection method is commonly used to introduce various types of protein and mRNA in cell lines and primary cells, but requires optimization according to the cell type to maintain viability but also to achieve efficient delivery. In terms of the applicability in other cell types, the electroporation step requires optimization, as well as the amount of Cas9 mRNA tolerated by the cell type, although it is very encouraging that this method is well tolerated in highly sensitive primary HSPCs. The components of the gene editing machinery itself have been shown to elicit immune, stress, and apoptotic responses^28^. While the colony-forming potential was not significantly affected by the editing procedure, we observed a reduction in in vivo engraftment compared to non-edited mock-electroporated cells, indicating that HSCs may be particularly sensitive to responses elicited by the gene editing, or that editing the CD45 locus specifically impairs transplantation^29^. Yet, overall, we achieved robust engraftment of CD45 edited cells, suggesting that the procedure is compatible with intact HSC potential and therefore suitable for studies of human HSC function.

## Methods

### Cloning

sgRNA sequences (Supplementary Table 3) were cloned into a modified version of the pLentiCRISPR v.2 vector (Addgene vector #52961, denoted pLCv2) following the U6 promoter, followed by the standard guide RNA backbone (chRNA1) or the modified guide RNA backbone (chRNA2), using BsmBI sites. In this modified vector, the Cas9-Puromycin resistance have been replaced by the EGFP fluorescent marker using restriction enzyme cloning. The chRNA2 backbone was cloned into the pLCv2-EGFP vector, replacing the chRNA1 backbone, by restriction enzyme cloning.

### Synthesis of Cas9 mRNA by in vitro transcription

Cas9 template DNA was isolated by digestion linearization of a pGEM-Cas9 vector, a kind gift from Dr. Donald B. Kohn laboratory, followed by PCR purification using GeneJET PCR purification kit (Thermo Fisher Scientific) according to the manufacturer’s protocol. The PCR product was then subjected to RNA synthesis using HiScribe T7 High Yield RNA Synthesis Kit (NEB) according to manufacturer’s instructions. The RNaeasy mini kit (QIAGEN) was then used for RNA purification according to manufacturer’s protocol. The purified product was then capped using Vaccinia Capping System (NEB) according to manufacturer’s instructions and purified again using the RNaeasy mini kit (QIAGEN). The concentration and purity of the RNA was measured using NanoDrop ND-1000 (Thermo Fisher Scientific). RNA was stored in − 80 °C until use.

### Primary human samples

Work with primary human samples was approved by the Regional Ethical Committee for Lund/Malmö (Regionala Etikprövningsnämnden i Lund/Malmö), approval #2010-696. All methods were carried out in accordance with relevant guidelines and regulations. CD34^+^ HSPCs were isolated from umbilical cord blood samples acquired from maternity wards of Helsingborg General Hospital and Skåne University Hospital in Lund and Malmö, Sweden. All samples were collected after informed consent. Mononuclear cells were separated through density-gradient centrifugation (Lymphoprep, Alere) and CD34^+^ HSPCs were enriched by magnetic bead purification (Miltenyi Biotec). For each cell batch used, CD34^+^ HSPCs from several cord blood units were pooled. Cells were stored in − 80 °C or − 150 °C until use.

### In vitro culture

K562 cells, kindly provided by Prof. Ewa Sitnicka Quinn, were maintained in HyClone RPMI-1640 Medium (GE Healthcare Life Sciences) supplemented with 10% heat-inactivated FBS (GE Healthcare Life Sciences) and 1% Penicillin–Streptomycin (GE Healthcare Life Sciences). CD34^+^ HSPCs were cultured in Serum-Free Expansion Medium (SFEM, STEMCELL Technologies) supplemented with 1% Penicillin–Streptomycin (GE Healthcare Life Sciences), stem cell factor (SCF), FLT3-ligand (FLT3L) and thrombopoietin (TPO). All cytokines were used in the concentration 100 ng/ml and acquired from Peprotech. CD34^+^ cells were thawed and pre-stimulated in culture medium at 37 °C, 5% CO_2_ for 48 h prior to RNP electroporation and for 24 or 48 h prior to lentiviral sgRNA transduction.

### Lentiviral production

Lentiviruses were produced in the human 293 T cell line (DSMZ, German Collection of Microorganisms and Cell Cultures) as described previously^30^.

### Lentiviral transduction

Transductions were carried out using RetroNectin (Takara Bio) following the RetroNectin-Bound Virus (RBV) Infection Method according to manufacturer’s protocol. Cells were transduced at a multiplicity of infection (MOI) of 10 with a target transduction efficiency of 40–50%. Cells were cultured at 37 °C, 5% CO_2_ for 24, 48, 72 or 96 h prior to electroporation.

### Electroporation of CB CD34^+^ hematopoietic stem and progenitor cells

For electroporation with Cas9/sgRNA RNPs, Cas9 protein (PNA Bio) in storage buffer and synthetic sgRNA (Synthego) in storage buffer (molar ratio 1:2.5) were incubated for 10 min in room temperature. Cas9 protein and Cas9 mRNA were thawed and incubated on ice until electroporation. 3 μg of Cas9 protein or 1.5 μg of Cas9 mRNA was used unless indicated otherwise. 80k–100k CB CD34^+^ cells were resuspended in BTXpress Electroporation Solution (Harvard Apparatus) and added to Cas9/sgRNA RNPs, Cas9 protein or mRNA to a total volume of 20 µl, which was then transferred to 1 mm cuvettes (Harvard Apparatus). Cells were immediately electroporated using the BTX ECM 830 electroporation system (Harvard Apparatus) with the following parameters set: 125 V, 950 µF, 5 ms, Ω none, Mode LV. The electroporated cells were allowed to recover for 10 min at room temperature. Cells were then cultured at 37 °C, 5% CO_2_ for 4 days prior to flow cytometry analysis and DNA extraction for NGS analysis. At this time point, cells were also counted using trypan blue to establish a baseline for single live cell normalization. For the transplantation experiment, both cell numbers and reagents were scaled up to 90 μl in the 1 mm cuvette. Cells from each electroporation cuvette were saved and kept in culture to be analysed by flow cytometry 4 days post-electroporation. Cells within the different treatment groups were pooled before transplantation into NSG mice.

### Flow cytometry

Electroporated CD34^+^ HSPCs were stained with CD45-AlexaFluor700 (BioLegend, clone H130), CD34-PE/Cy7 (BioLegend, clone 581), CD44-BV421 (BioLegend, clone IM7), and CD29-APC (BD Pharmingen, clone MAR4 (RUO)). Mouse BM cells were stained with CD45-AlexaFluor700 (BioLegend, clone H130), HLA-A,B,C-PE/Cy7 (BioLegend, clone W6/32), CD33-PE (BioLegend, clone WM53), and CD19-BV605 (BD Biosciences, clone SJ25C1). All antibodies were used in the concentration recommended by the manufacturer. Dead cells and debris were excluded by forward scatter (FSC), side scatter (SSC), DAPI or 7-AAD staining (1:100 dilution prior to analysis). The flow cytometry analyses were performed with BD LSR II instrument (BD Biosciences) or BD LSRFortessa instrument (BD Biosciences). To analyse the data, the software FlowJo (FlowJo, LLC) was used.

### Next generation sequencing

DNA extraction was performed using QIAamp DNA Blood Mini Kit (QIAGEN) according to manufacturer’s protocol. The genomic target sequence was amplified using the Q5 Hot Start High-Fidelity 2X Master Mix (NEB) or the Phusion High-Fidelity PCR Master Mix with HF Buffer (Thermo Fisher Scientific) with primers designed for the target gene (Supplementary Table 4). PCR reactions were carried out on a T100 Thermal Cycler (Bio-Rad) following manufacturer’s protocol. The PCR protocol involved one cycle of 98 °C (4 min), 34 cycles of 98 °C (30 s), 68 °C (1 min), 72 °C (3 min) and one cycle of 72 °C (2 min). The PCR products were run on a 1.5% agarose gel with GelRed Nucleic Acid Stain (Biotium). Bands for the specific products were cut out. Gel extraction was performed using GeneJET Gel Extraction Kit (Thermo Fisher Scientific) according to manufacturer’s protocol. The purified DNA was subjected to tagmentation followed by a secondary indexing PCR according to manufacturer’s instructions using Nextera XT DNA Library Preparation Kit (Illumina) and Nextera XT Index Kit v2 Set A indexing kit (Illumina). Prepared samples were then sequenced on the NextSeq 550 System or the MiSeq System (Illumina). Insertion/deletion (Indel) frequencies were quantified using an in-house algorithm, TIGERq, to calculate the raw gene editing efficiency. When edited sequenced samples were partially transduced with EGFP tracking constructs, raw gene editing efficiency scores were divided by the percent EGFP^+^ cells to provide an estimate of editing efficiency within the transduced population.

### Off-target analysis

Off-target (OT) sites were designed using the computational software COSMID^31^. Predicted off-target sites were ranked by their COSMID score, with OT sites sharing location on top of the CD45 sg5 spacer (due to indels) were excluded. The remaining top 4 ranked OT sites were used for NGS. Off-target NGS was carried out as described for the on-target site sequencing and analysis. Variants that did not pass filter for number of events detected (20 events cutoff per variant), or that did not pass filter for broad read range contribution (variant reads must originate from multiple positions) were excluded. Analyzed percent modification was normalized to on-target percent modification to account for higher off target activity from better on-target performing sgRNAs.

### Methylcellulose colony-formation assay

To assess the frequency of colony-forming cells (CFCs), the colony-forming unit (CFU) Assay was performed by FACS sorting single, live (7AAD^-^) CD34^high^EGFP^high^ and/or CD45^-^ cells into methylcellulose (MethoCult H4230, STEMCELL Technologies) supplemented with SCF (25 ng/ml), GM-CSF (50 ng/ml), IL-3 (25 ng/ml) (Peprotech) and EPO (5U/ml, Apoteket) 3 days post-electroporation (7 days in culture). Plates were incubated at 37 °C, 5% CO_2_ and mature hematopoietic colonies were assessed after 13 days.

### Human engraftment assay

Transduced non-electroporated, transduced mock-electroporated or transduced Cas9-electroporated bulk CD34^+^ cells were injected into the tail vein of sublethally irradiated (3 Gy) NOD Cg-Prkdc^scid^ Il2rg^tm1Wjl^/SzJ (NSG) mice one day post-electroporation. Mice were euthanized after 9–12 weeks and BM was harvested for analysis of human engraftment and editing efficiency. All animal experiments were approved by the local Ethical Committee (Malmö-Lunds Djurförsöksetiska Nämnd) approval# 9636–18. All methods were carried out in accordance with relevant guidelines and regulations.

### Statistical analysis

Statistical significance was calculated in GraphPad Prism 7 (GraphPad Software) using unpaired student’s t-test, paired student’s t-test, Two-way ANOVA, or Tukey’s multiple comparisons test. Error bars represent standard deviation (s.d.) unless indicated otherwise.

## Supplementary Information

Supplementary Information.

## Data Availability

The datasets generated during and/or analysed during the current study are available from the corresponding author on reasonable request.
