# Supplementary material for: Combined lentiviral- and RNA-mediated CRISPR/Cas9 delivery for efficient and traceable gene editing in human hematopoietic stem and progenitor cells
Source: Sci Rep. 2020 Dec 28;10:22393. doi: 10.1038/s41598-020-79724-x (PMC7769964; doi:10.1038/s41598-020-79724-x)
Supplement: Supplementary file 1 — Supplementary Information. [file 41598_2020_79724_MOESM1_ESM.pdf]

Supplementary Information

Combined Lentiviral- and RNA-mediated CRISPR/Cas9 Delivery for Efficient and Traceable Gene Editing in Human Hematopoietic Stem and Progenitor Cells

David Yudovich[1,2], Alexandra Bäckström[1,2], Ludwig Schmiderer[1], Kristijonas Žemaitis[1], Agatheeswaran Subramaniam[1] and Jonas Larsson[1*]

[1]Division of Molecular Medicine and Gene Therapy, Lund Stem Cell Center, Lund University, Lund, Sweden
[2]Equal contribution

*Correspondence to Jonas Larsson MD PhD, Molecular Medicine and Gene Therapy, BMC A12, 221 84, Lund, Sweden; phone: +46-46-2220580; fax: +46-46-2220568;
e-mail: jonas.larsson@med.lu.se

Supplementary Table 1. List of unique predicted off-target sites for CD45 sg5

Number	Chr position	Score	NGS
1	Chr2:28427250-28427271	0.97	Yes
2	Chr8:144448492-144448513	0.99	Yes
3	ChrX:153573824-153573845	2.43	Yes
4	Chr6:42935481-42935501	3.02	Yes
5	Chr15:90000801-90000821	3.02	PCR failed
6	Chr16:49494734-49494754	4.68	N/A
7	Chr7:98949526-98949547	4.7	N/A
8	Chr16:48447603-48447623	5.64	N/A
9	Chr18:76612153-76612173	5.86	N/A
10	Chr19:8903492-8903513	6.13	N/A

Supplementary Table 2. Off-target editing (% modification normalized to on-target efficiency for each condition) for CD45 sg5

	**Off-target site**			
**Sample**	**#1** Chr2:28427250-28427271	**#2** Chr8:144448492-144448513	**#3** ChrX:153573824-153573845	**#4** Chr6:42935481-42935501
Untreated	0.03	0.00	0.00	0.00
RNP (3 µg)	0.00	10.15	1.91	1.16
mRNA (1.5 µg)	0.58	4.47	0.63	0.29
LV sg5 + protein (3 µg)	0.00	0.00	0.00	0.00
LV sg5 + mRNA (1.5 µg)	0.13	2.00	0.00	0.00

Supplementary Table 3. sgRNA sequences

Gene target	Guide	Sequence
CD45	sg5*	GGTGCTGGTGTTGGGCGCAC
CD45	sg8	GGGCGCACAGGAACCTATAT
Non-targeting	ntA	GACGGAGGCTAAGCGTCGCAA
CD44	sg1	GAAGGGCACGTGGTGATTCCC
CD44	sg2	GCAATATGTGTCATACTGGG
CD44	sg3	GTACAGGTATGGGTTCATAGA
CD44	sg4	GCCAGCTATTGTTAACCGTGA
CD29	sg1	GTCATCACATCGTGCAGAAGT
CD29	sg2	GAATGTAACCAACCGTAGCAA
CD29	sg3	GCATAAGGTAGTAGAGGTCAA
CD29	sg4	GACAGCGCATATCTGGAAATT

*sgRNA sequence adapted from Gundry *et al.* 2016.

Supplementary Table 4. Primer sequences

Primer	Sequence 5'-3'
CD45_G5_Tag_FWD	CTGGGGCTGACTTTCCTCAG
CD45_G5_Tag_REV	TGAGCATCTCTCCAAACAGACA
CD44_G1t3_Tag_FWD	GTGGGGGAATGGGACATGAG
CD44_G1t3_ Tag _REV	GCACTTTCCCTGACCACCTT
CD44_G4_ Tag _FWD	TCCTAGCCAGAAGTGGGTGT
CD44_G4_ Tag _REV	AGGAGGGCTCTCCCCAAAAT
CD29_G1t2_ Tag _FWD	AGCAGGATGAGGAGGAGAGG
CD29_G1t2_ Tag _REV	ACACTTGGGTCAGTTCTGGG
CD29_G3_ Tag _FWD	TTGGCACACCAGCTCTCAAT
CD29_G3_ Tag _REV	GTGGCATCCCACTACACACA

Supplementary Figure S1

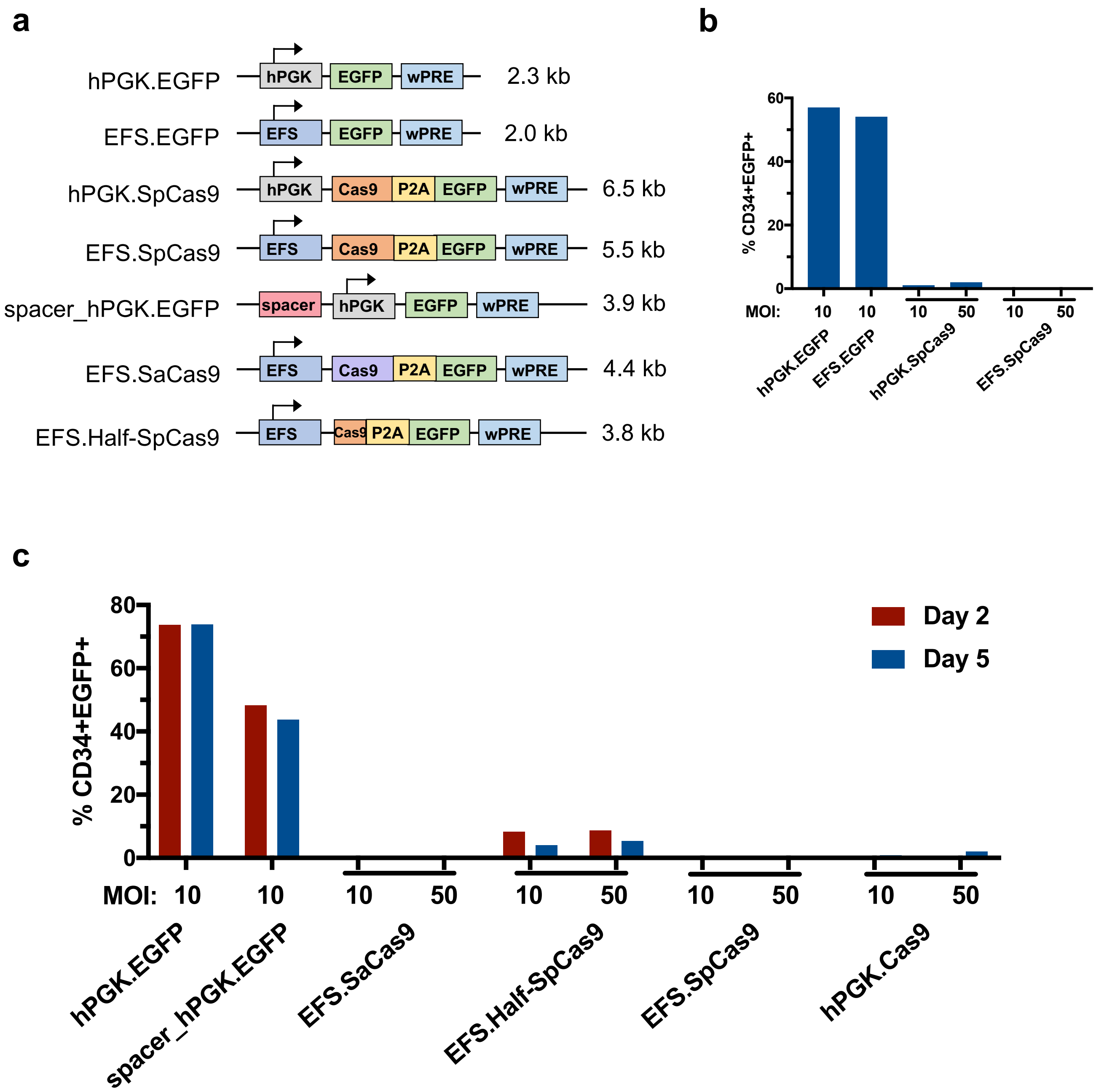

Supplementary Figure S1. Challenge of lentiviral Cas9 delivery in human CD34⁺ HSPCs

(**a**) Schematic outline of pLCv2 vectors. (**b and c**) CD34⁺ HSPCs were transduced on day 2 post-thaw. EGFP and CD34 expression was analysed by flow cytometry on day 2 and/or day 5 post-transduction.

Supplementary Figure S2

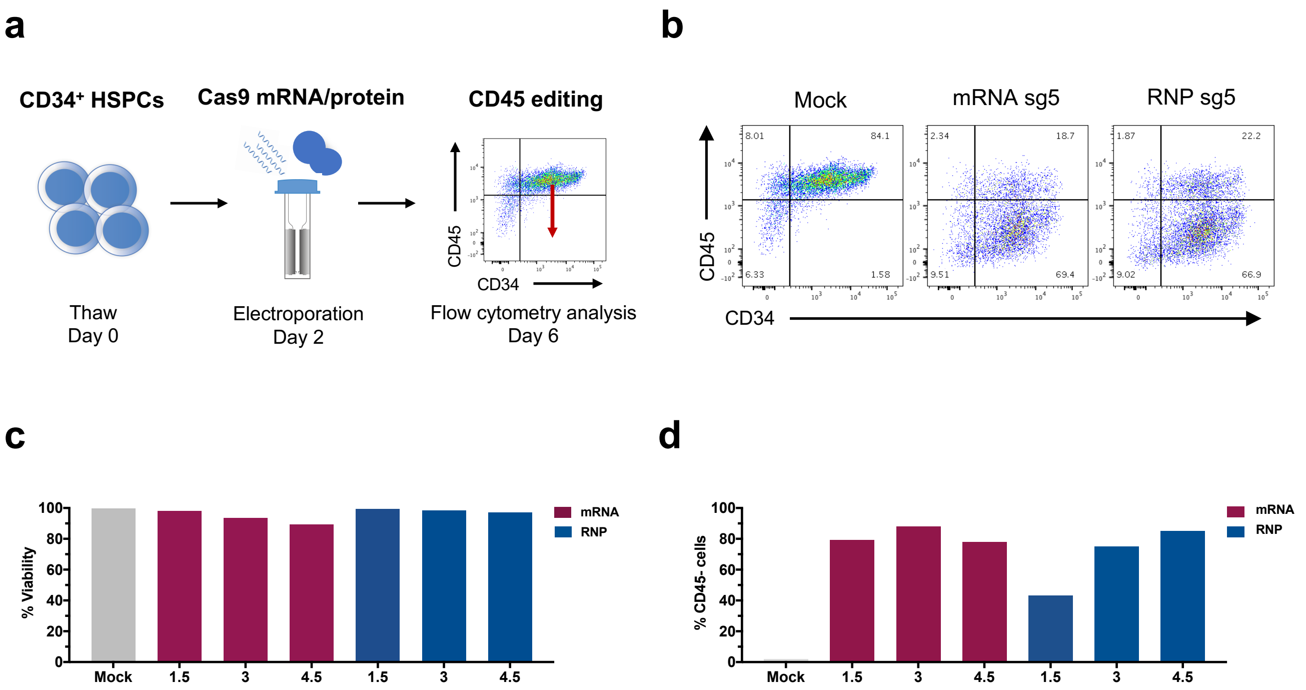

Supplementary Figure S2. CD45 editing using Cas9 mRNA or protein with synthetic, modified sgRNA

CD34⁺ HSPCs were treated with Cas9 mRNA or protein at various concentrations, in combination with co-electroporated modified, synthetic sgRNA targeting CD45 (sg5), and analysed by flow cytometry. (**a**) Overview of experimental outline. (**b**) Representative FACS plots of CD45 editing using 1.5 µg Cas9 mRNA or 3 µg Cas9 protein. (**c**) Viability analysis by flow cytometry 7AAD staining. (**d**) Frequency of CD45 edited cells.

Supplementary Figure S3

a

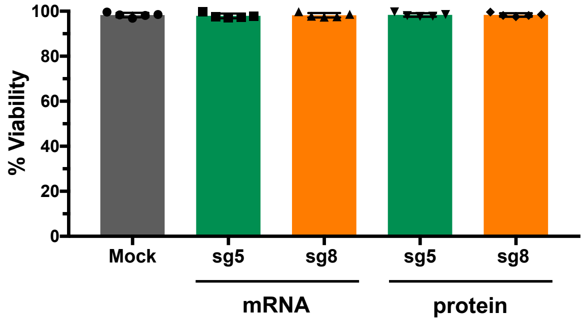

b

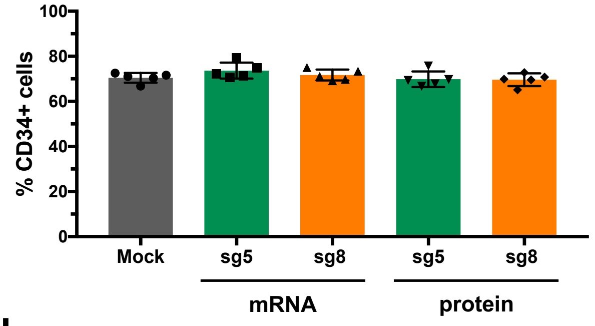

c

Sample	Flow cytometry negative cells (%)	NGS editing raw value (%)	NGS editing adjusted to EGFP (%)
Mock	1.7	0.0	0.0
mRNA sg5	47.1	15.3	38.2
mRNA sg8	21.6	10.0	23.8
Protein sg5	10.0	11.3	21.8
Protein sg8	2.1	0.3	0.7

d

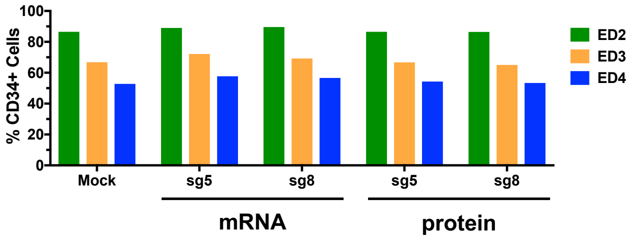

e

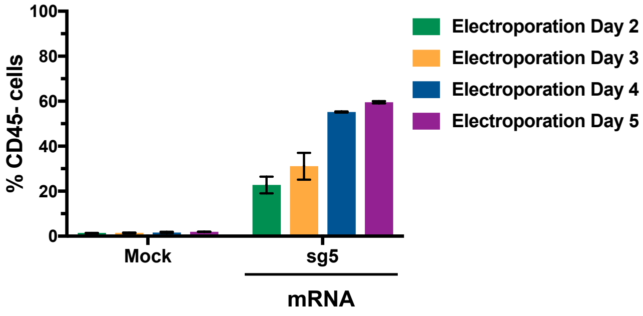

Supplementary Figure S3. Effect on viability and CD34 expression from HSPC editing using Cas9 mRNA or protein with lentiviral sgRNA

(**a and b**) Primary $CD34^+$ HSPCs were transduced with sgRNA targeting CD45 (sg5 or sg8) and subsequently electroporated with Cas9 mRNA or protein (n=5). (**a**) Viability measured by 7AAD staining, (**b**) $CD34^+$ expression analysed by flow cytometry 4 days following the electroporation and (**c**) NGS analysis for representative samples, cells were collected 4 days following the electroporation. Note, raw gene editing efficiency scores were divided by percent $EGFP^+$ cells to provide an estimate of editing efficiency within the transduced population. (**d**) $CD34^+$ cells were transduced on day 1 with sgRNA targeting CD45, electroporated on either day 2, 3 or 4, and subsequently analysed by flow cytometry for CD34 expression 4 days following the electroporation. (**e**) $CD34^+$ cells were transduced on day 1 with sgRNA targeting CD45, electroporated with Cas9 mRNA on either day 2, 3, 4 or 5, and subsequently analysed by flow cytometry for CD45- cells 4 days following the electroporation.

Supplementary Figure S4

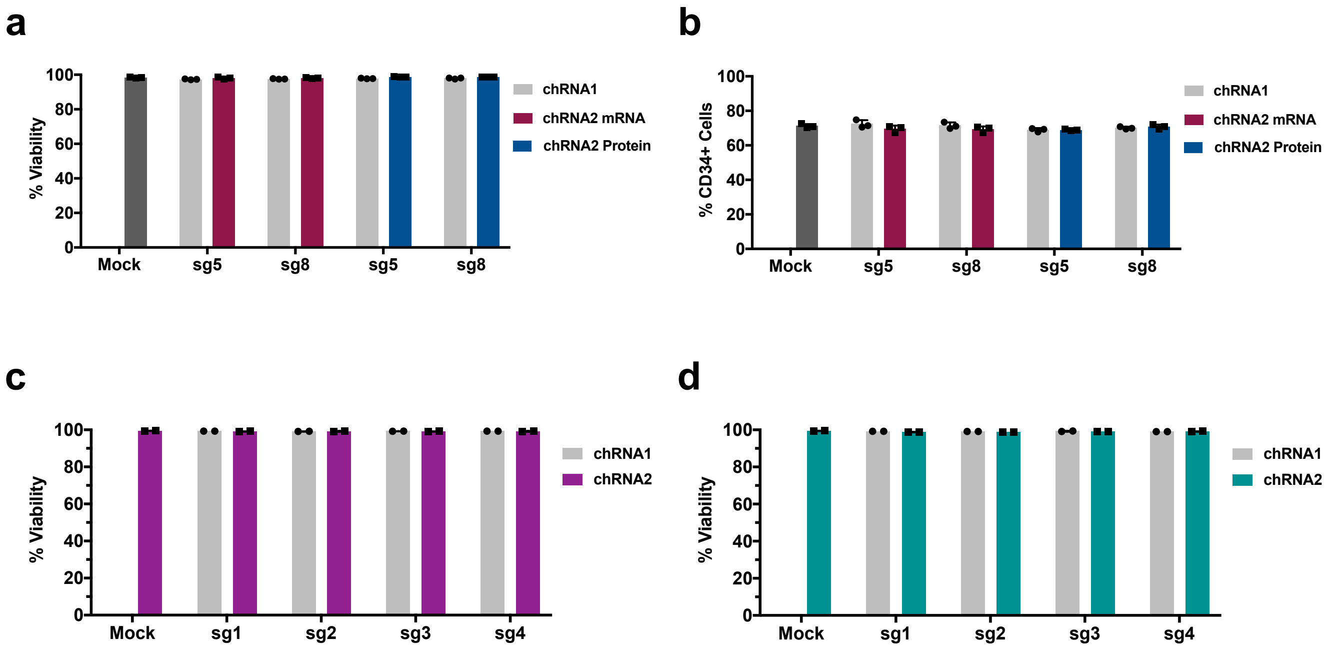

e

Sample CD44	Flow cytometry negative cells (%)	NGS editing raw value (%)	NGS editing adjusted to EGFP (%)
Mock	5.9 ± 1.5	0.0 ± 0.0	0.0 ± 0.0
sg3 chRNA1	22.3 ± 3.7	17.7 ± 3.8	32.1 ± 6.6
sg3 chRNA2	56.8 ± 14.8	36.6 ± 15.6	67.5 ± 30.4
sg4 chRNA1	76.6 ± 2.8	43.2 ± 14.4	95.8 ± 5.9
sg4 chRNA2	87.2 ± 1.7	51.7 ± 2.5	100 ± 0.0

f

Sample CD29	Flow cytometry negative cells (%)	NGS editing raw value (%)	NGS editing adjusted to EGFP (%)
Mock	5.9 ± 1.5	0.0 ± 0.0	0.0 ± 0.0
sg1 chRNA1	53.5 ± 3.7	33.2 ± 1.6	91.4 ± 2.2
sg1 chRNA2	61.5 ± 1.2	41.6 ± 7.4	91.7 ± 11.7
sg3 chRNA1	30.8 ± 10.2	21.9 ± 5.7	54.9 ± 13.7
sg3 chRNA2	61.4 ± 17.6	42.9 ± 9.3	87.3 ± 18.0

Supplementary Figure S4. Modified chimeric guide RNA backbone enhances editing efficiency in HSPCs without compromising viability or CD34 expression
CD34$^+$ cells were transduced with sg5 or sg8 targeting CD45 encoding either chimeric guide RNA backbone 1 or 2 on day 1 post-thaw, electroporated with Cas9 mRNA or protein on day 4, and analysed by flow cytometry on day 8 (n=3). (**a**) Viability by 7AAD staining and (**b**) CD34$^+$ expression.
CD34$^+$ cells were transduced on day 1 post-thaw with 4 different lentiviral sgRNAs per target gene encoding either chimeric guide RNA backbone 1 or 2, electroporated with 2 μg Cas9 mRNA on day 4, and analysed by flow cytometry on day 8. At this time point, cells were also collected for NGS (n = 2). (**c**) Viability by 7AAD staining in CD44 edited cells and (**d**) in CD29 edited cells. (**e**) NGS analysis of the CD44 locus and (**f**) of the CD29 locus in edited cells ± standard deviation (s.d.). Note, raw gene editing efficiency scores were divided by percent EGFP$^+$ cells to provide an estimate of editing efficiency within the transduced population.

Supplementary Figure S5

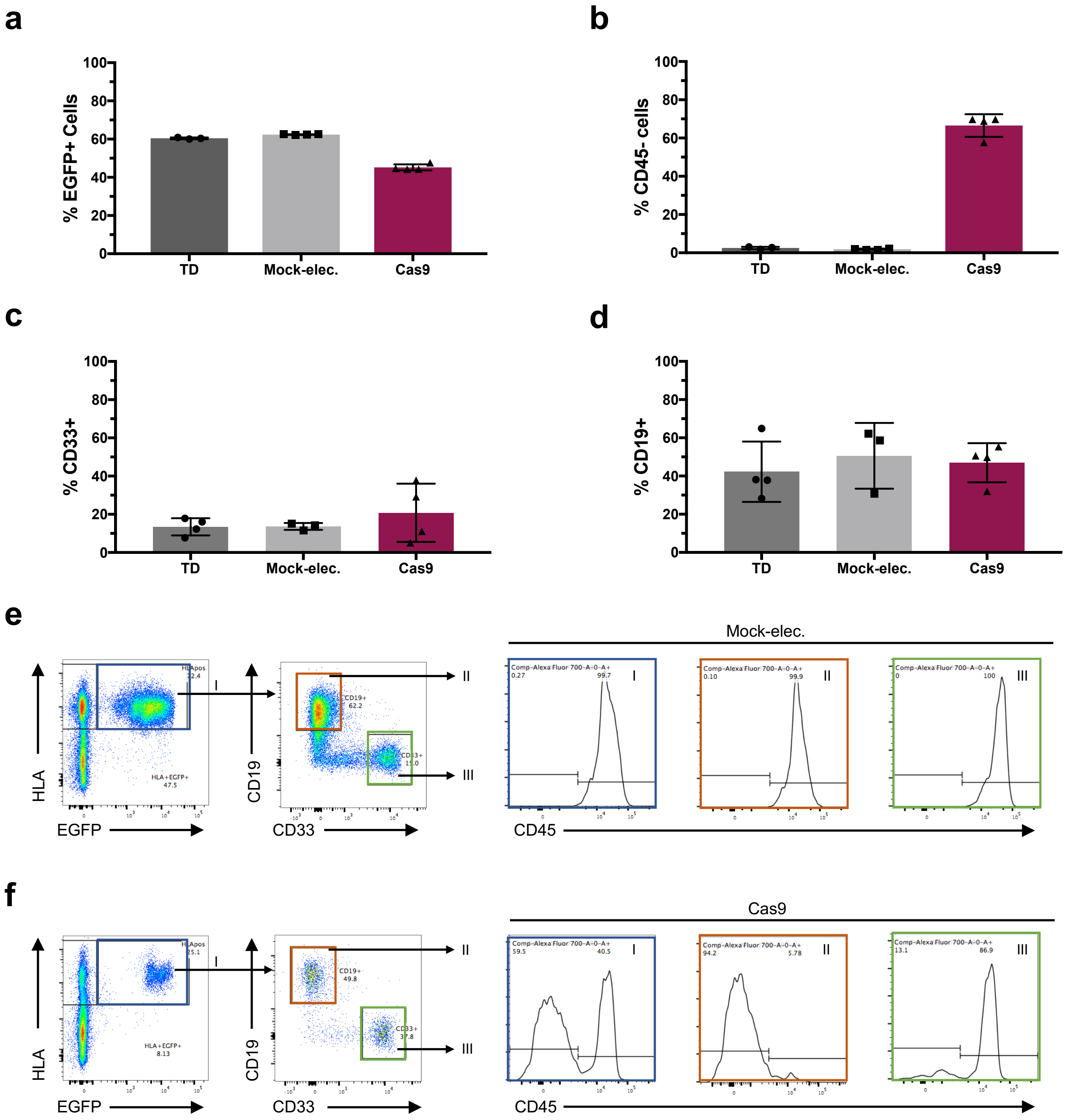

Supplementary Figure S5. Edited CD34^{+} HSPCs retain reconstitution capacity in vivo (**a and b**) A portion of edited CD34^{+} cells from each group was not used for transplantation and maintained in culture and analysed by flow cytometry 4 days after electroporation. (**a**) Frequency of EGFP^{+} cells. (**b**) Frequency of CD45- cells in the EGFP^{+} population. (**c-f**) BM from transplanted NSG mice harvested 9-12 weeks post-transplantation analysed by flow cytometry. n=3-4 mice per group. (**c**) Frequency of CD33^{+} cells within the HLA^{+}EGFP^{+} population. (**d**) Frequency of CD19^{+} cells within the HLA^{+}EGFP^{+} population. (**e and f**) Gating strategy to determine CD45 editing in the HLA^{+}EGFP^{+} population (I), CD19^{+} population (II) and in the CD33^{+} population (III), showing examples for (**e**) mock-electroporated and (**f**) Cas9-electroporated cells.
